# Sequential Processing of Cold Gas Sprayed Alloys by Milling and Deep Rolling

**DOI:** 10.3390/ma14133699

**Published:** 2021-07-01

**Authors:** Daniel Meyer, Lars Schönemann, Nicole Mensching, Volker Uhlenwinkel, Bernhard Karpuschewski

**Affiliations:** 1 University of Bremen and MAPEX Center for Materials and Processes, Bibliothek Str. 1, 28359 Bremen, Germany; dmeyer@iwt-bremen.de (D.M.); schoenemann@iwt.uni-bremen.de (L.S.); uhl@iwt.uni-bremen.de (V.U.); karpuschewski@iwt-bremen.de (B.K.); 2 IWT Leibniz Institute for Materials Engineering, Badgasteiner Str. 3, 28359 Bremen, Germany

**Keywords:** cold gas spraying, deep rolling, milling, plastic deformation, surface properties

## Abstract

Cold gas spraying (CS) is a solid-state material deposition process which, in addition to the flexible repair of individual component areas, also enables the build-up of larger samples. The layers are created on a substrate by the impact-induced bonding of highly accelerated micrometer particles. Since melting does not occur, the material composition can be varied flexibly and independently of material-specific melting points. In this work, the influence of the described forming process on subsequent machining by milling and deep rolling is investigated. The process forces measured during milling and the surface topography after milling and deep rolling were influenced by the material composition and the CS-related properties, e.g., high material hardness or particle bonding. In contrast to prior assumptions, deep rolling was shown to have no influence on the determined hardness depth profile for the investigated materials. Future work will focus on additional analyses, such as the determination of half-widths, to obtain further insight on the material behavior.

## 1. Introduction

The possibility of fast and application-oriented manufacturing of highly complex components has led to the increased importance of Additive Manufacturing (AM) processes in multiple industrial sectors, such as medical technology or aerospace [1]. By offering new design options, AM enables near-net-shape production and, thus, a weight-reduced component design [2]. Various AM techniques such as Laser Powder Bed Fusion (LPBF) or Laser Metal Deposition (LMD) have been developed within recent years and are applied to generate parts made of metal, polymers, and composites in a layer-based way [3]. Most AM processes involve the melting of the material during the generation of the component, which results in high temperature gradients and complex residual stress distributions [4]. Crack formation, material oxidation, or undesired phase transformation may occur. During the melting process, the properties and composition of the supplied powder are changed in laser-based AM.

Within this work, three different cold gas sprayed (CS) alloys are investigated (316L, FeTiB and FeMnAlC). CS is a method originally developed as a coating technology [5,6] and is increasingly gaining importance in the field of additive manufacturing [7]. It involves a low-temperature gas jet accelerating particles with micrometer-scale sizes (powders) accumulating into dense coatings due to severe plastic deformation when impacting on a substrate [8,9,10]. According to Yin et al., this severe plastic deformation is the basic requirement for intermixing at the substrate–coating interface, which influences the adhesion of the coating [11]. Depending on the process design, a distinction is made between high-pressure cold spray and low-pressure cold spray, resulting in lower or higher particle velocities, respectively [7]. According to the review of Rokni et al., the extent of particle deformation and, thereby, also the deposition efficiency is influenced by the particle temperature and particle velocity [7]. The particle velocity needs to exceed a critical velocity to adhere on the substrate [12]. This critical velocity is influenced not only by the material of the particle and the substrate but also by, e.g., the processing parameters, the particle size and the topological properties of the substrate surface [13]. Since the particles bond due to their kinetic energy, no powder melting and, thus, evaporation-related changes of the alloying composition usually occur [13]. Thus, CS is suitable for temperature-sensitive or oxygen-sensitive materials [14,15]. Furthermore, it allows the combination of different materials already in powder form, which, as shown by AL-Mangour et al. using 316L and Co-Cr, not only improves tensile strength but also corrosion properties compared to pure stainless steel [16]. Considering the microstructure, the large strains (up to 10) and strain rates (up to 10^9^ s^−1^) lead to work hardening, which, together with the severe plastic deformation, temperature increase, and intense shear stress, in turn leads to grain refinement by dynamic restoration phenomena (dynamic recovery and dynamic recrystallization) at the particle–substrate and particle–particle interfaces [7,17]. According to Ogawa et al., the intense particle deformation during CS can further lead to a loss of ductility [18], which can be improved, for example, by post-CS heat treatment [7].

Regardless of the applied coating or AM process, the generated parts usually show drawbacks such as lower tensile strength, porosity or a high surface roughness, which in most applications requires additional post-processing by, e.g., heat treatment [7,16], hot isostatic pressing (HIP), milling or turning. In some cases, the cutting process is followed by a mechanical surface treatment [19] such as deep rolling, shot peening [20] or machine hammer peening [21] to further improve the surface roughness and to introduce strain hardening as well as compressive residual stresses [22]. Although these processes lead to an extension of the process chain, they are generally accompanied by an improvement in fatigue strength and, therefore, an extended component lifetime [23,24,25].

This has been investigated for LPBF components, for example by Breidenstein et al., who performed experiments on the influence of milling and deep rolling on the Surface Integrity of H13-steel-parts [22]. Meyer and Wielki also investigated the potential of deep rolling as a finishing process of AM steel parts and showed that improvements in surface roughness and subsurface properties are possible even without an intermediate cutting process [26,27]. However, corresponding investigations for CS samples have not yet been carried out systematically.

In this work, three cold gas sprayed alloys (316L, FeTiB and FeMnAlC; coating thickness > 2 mm) are post-processed by milling and/or deep rolling to develop procedures for the post-processing of future standalone parts (detached from the substrate) based on the analyzed surface and subsurface properties. While 316L is a commercially available and established alloy, the other two alloys have not yet been considered in the CS process. With the aim of a rapid usability of the new powders, a time-consuming parameter study is deliberately dispensed with and the potential of deep rolling to overcome the drawbacks of non-optimized CS parameters is investigated. Both the as-sprayed surface as well as the processed surfaces are investigated. By varying the process parameters, the machinability of the cold gas sprayed alloys is assessed. Furthermore, the influence of the processing steps on the subsurface properties is investigated by aid of micrographs, hardness depth profiles, and EBSD measurements. Aiming at the mentioned compensation of drawbacks due to the parameter settings not being material-adjusted, the aim of the current work is to find out to what extent the plastic deformation associated with the CS can be further influenced by deep rolling.

## 2. Materials and Methods

### 2.1. Cold Gas Sprayed Coatings

Within this work, cold gas spraying of three steel alloys (316L, FeTiB and FeMnAlC) was investigated. Samples were generated by an Impact Spray System 5/11 (Impact Innovations GmbH, Rattenkirchen, Germany) at the test center of the manufacturer. The chemical composition of the particles can be found in Table 1. While the 316L powder is commercially available (supplier: Sandvik, Sandviken, Schweden, cf. [28]) and widely applied in AM, the FeMnAlC and the FeTiB powder was generated by inert gas atomization at the Leibniz Institute for Materials Engineering (Bremen, Germany).

Cold spraying was performed using aluminum and steel substrates (size: 5 mm × 50 mm × 30 mm). While the aluminum substrates were cleaned using ethanol, the steel substrates were sand blasted before cold gas spraying. Table 2 summarizes the cold gas spraying parameters. It has to be noted that these parameters have been selected according to the recommendations of the aforementioned producer of the spray system and are optimized for spraying 316L. Due to the limited experience in cold spraying FeTiB and FeMnAlC, the settings may not yet be optimal for these alloys. As a consequence, the deposition efficiency of the coatings varies from approx. 94.9% for 316L to 77.6% for FeMnAlC and 18.7% for FeTiB. The thickness of the material layered by means of cold gas spraying is 2.1 mm for 316L, 2 mm for FeMnAlC and 2.5 mm for FeTiB.

With a gas flow of 4 m^3^/h, a disc speed of 3 rpm (disc type: 120) and an open bypass, powder was supplied through the first powder feeder. The second powder feeder was used for cooling (water: 10 °C).

### 2.2. Post Processing

#### 2.2.1. Milling

The machinability of the cold sprayed samples in an as-sprayed state was analyzed by milling individual tracks and determining the cutting forces. Milling experiments were performed on an Ultrasonic 20 linear milling machine (DMG Mori, Bielefeld, Germany).

**Figure 1:**
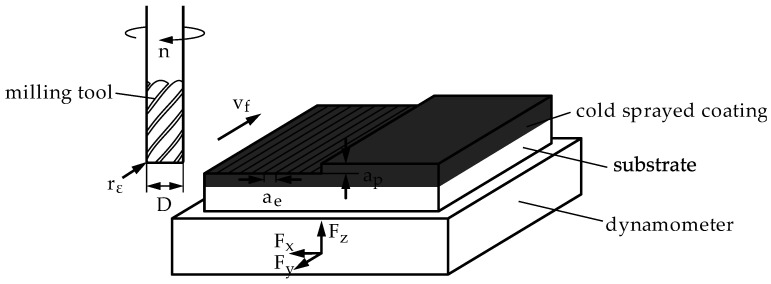
Experimental setup for the milling experiments.

Figure 1 shows the experimental setup. A toroidal cutter with a diameter of D = 12 mm, six teeth (z_t_ = 6), and a corner radius of r_c_ = 0.5 mm was used. While the spindle speed n (8000 min^−1^), the cutting speed v_c_ (302 m∙min^−1^), the depth of cut a_p_ (1 mm), and the width of cut a_e_ (0.6 mm) were kept constant, the feed velocity v_f_ was varied between 2800 mm∙min^−1^ and 4600 mm∙min^−1^ in steps of 200 mm∙min^−1^, i.e., with a feed per tooth of f_z_ ≈ 58.3 µm to 95.8 µm in steps of 4.2 µm. Experiments were repeated 3 times for a basic assessment of reproducibility. Cutting forces were measured utilizing a piezoelectric 3-component dynamometer (Typ 9256C 2, Kistler Instrumente GmbH, Sindelfingen, Germany) with a sampling rate of 30 kHz. Within this work, the active force F_a_ of the X- and Y force components (F_x_, F_y_) was calculated and used as the basis for all comparisons (Equation (1)):

(1) $$F_{a}=\sqrt{F_{x}^{2}{+F}_{y}^{2}}$$

For each experiment, the mean of F_a_ and the standard deviation of the mean s_Fa_ was determined by averaging the peak force values during stable cutting conditions, which occur at a frequency of f_peak_ = n/60⋅z_t_ = 800 Hz. Thereafter, the results of all three experiments were merged by calculating the weighted mean and its error.

For surface analysis, the Keyence digital microscope VHX—S 15 (KEYENCE DEUTSCHLAND GmbH, Neu-Isenburg, Germany) and the coherence scanning interferometer Talysurf CCI HD (AMETEK GmbH, BU Taylor Hobson, Weiterstadt, Germany) were used, enabling high resolution imaging as well as the three-dimensional profile measurements of structures and surfaces in the range of a few micrometers.

#### 2.2.2. Deep Rolling

**Figure 2:**
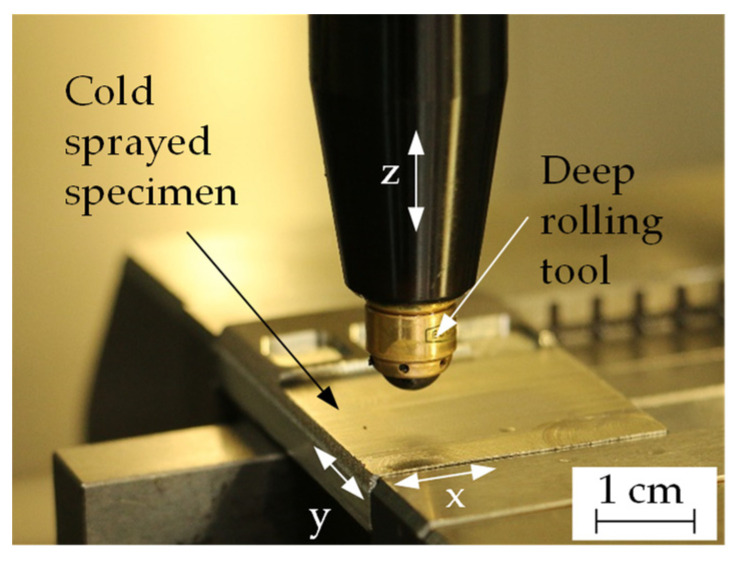
Experimental setup for deep rolling.

Deep rolling experiments were performed on a 3-axis CNC machining center (DMC 65V by Deckel Maho, Pfronten, Germany) utilizing a hydrostatic deep rolling tool with a spherical ceramic tip (diameter: 6 mm) Ecoroll HG6 (Ecoroll AG Werkzeugtechnik, Celle, Germany). The experimental setup is shown in Figure 2.

Table 3 lists the process parameters applied in the deep rolling experiments. The deep rolling force F_r_ was measured utilizing a piezoelectric 3-component dynamometer (Typ 9257 B, Kistler Instrumente GmbH, Sindelfingen, Germany) equipped by a Kistler charge amplifier Typ 5019 A (low pass filter: 500 Hz; sampling rate: 2500 Hz).

**Figure 3:**
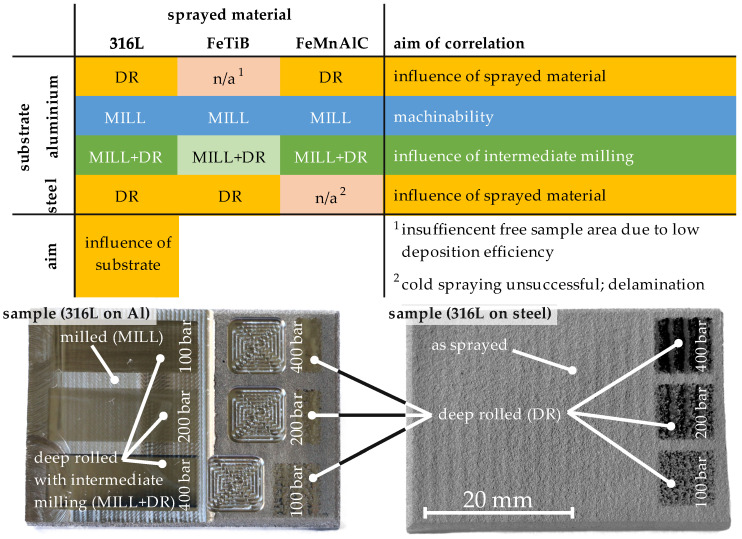
Schematic illustration of the experimental procedure.

Furthermore, cross-sections of the processed areas were generated to gain further insights about the microstructure of the investigated cold gas sprayed samples and the influence of the applied post-processing strategies on the microstructure. For selected samples, these are supported by hardness depth profiles. Figure 3 shows an overview of the experiments performed on each sample.

### 2.3. Further Devices

The hardness values discussed in this paper were determined in conformity with the standard according to Rockwell (HRC) with a cone-shaped diamond indenter and according to Vickers with an equal-sided diamond pyramid indenter. EBSD measurements were performed on a Helios G4 PFIB CXe (Thermo Fisher Scientific, 168 Third Avenue Waltham, MA USA 02451). With increments of 0.1 µm, measuring fields of a size of 50 × 100 µm^2^ were acquired (voltage: 20 kV, current: 6.4 nA). The image quality (IQ), the inverse pole figure (IPF), and the Kernel Average Misorientation (KAM) were determined.

## 3. Results

In this section, the initial state of the alloys after cold spraying is presented first. For a direct comparison, in addition to the micrographs shown, hardness measurements according to Rockwell were carried out. The results of the experimental program are first presented for the milling process. These are followed by the results obtained after deep rolling with different deep rolling pressures with and without intermediate milling.

### 3.1. Inital State of CS Coatings

**Figure 4:**
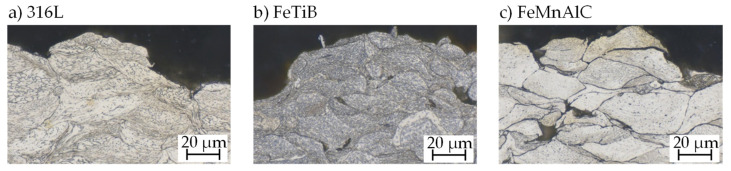
Microstructure of the cold sprayed samples: (**a**) etched 20 s in 60% electrolytic HNO_3_ (1.5 V); (**b**,**c**) etched 15–20 s in 3% alcoholic HNO_3_.

While 316L and FeTiB exhibited a good bonding behavior in both cases, usable coatings with FeMnAlC could only be generated on the aluminum substrate. The hardness of the applied coatings was measured to 34 HRC for 316L, 37 HRC for FeMnAlC, and 52 HRC for FeTiB. Figure 4 shows the microstructure of the as-sprayed surface. Cross-sections were etched for 15 s (FeTiB) or 20 s (FeMnAlC) using 3% alcoholic HNO_3_ and for 30 s using 60% electrolytic etchant. The high surface roughness directly after cold gas spraying becomes obvious in the micrographs. Individual bonded particles can be identified and pores are visible for FeTiB and FeMnAlC.

### 3.2. Milling

**Figure 5:**
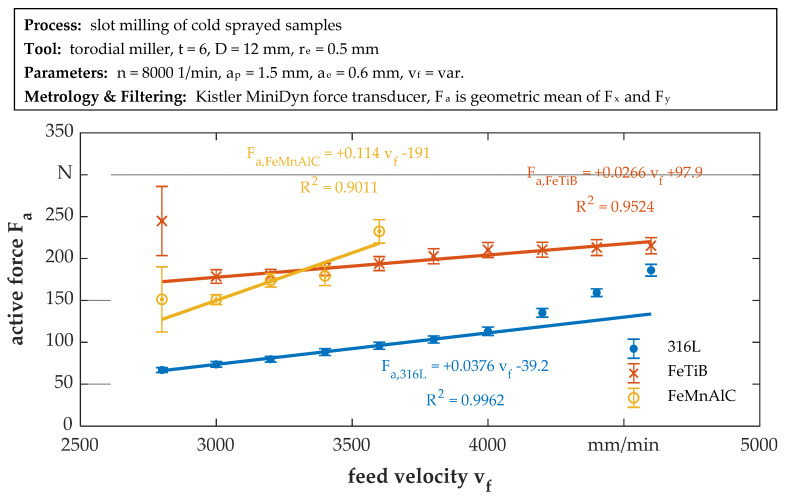
Feed velocity dependent active cutting force; force values are the weighted mean of F_a_ for three experiments, error bars indicate the error of the mean as the maximum of the internal (error within each measurement) and external (error across the repetitions) error.

To evaluate the machinability of the cold-sprayed samples, measurements of the cutting forces during milling were performed. Figure 5 shows the active force F_a_ plotted over the feed velocity v_f_ for each material. A systematic error is present for the initial values (v_f_ = 2800 mm/min) for FeTiB and FeMnAlC, which is indicated by a large (external) error. In these two cases, the three repetitions of the experiment were consistent within themselves (low internal error), but did not yield similar force levels. This may be attributed to unequal cutting conditions, as this was the first cut into the substrate. The cutting strategy was changed for 316L, which therefore shows no systematic error for the initial run. As the feed rate increases, the active force increases for all materials, as can be expected, and a (weighted) linear fit of the progression yields high determination coefficients if outliers are disregarded (v_f_ = 2800 mm/min for FeMnAlC and FeTiB and v_f_ > 4000 mm/min for 316L). The active forces for 316L are lower than those of the other materials for all parameters. The active force for FeMnAlC starts at a medium level around 150 N but shows the strongest increase thereafter (max. value: F_a_ ≈ 232.5 N at v_f_ = 3600 mm⋅min^−1^). This increase was so significant that the experiments had to be stopped because the selected measurement range of the dynamometer (0–250 N per channel) was exceeded. Active forces for cutting FeTiB start at the highest initial value of all alloys (F_a_ ≈ 178.5 N at v_f_ = 3000 mm/min, resp. F_a_ ≈ 245 N at v_f_ = 2800 mm/min, which is considered as an outlier), but only gradually increase with feed velocity (max. value: F_a_ ≈ 220 N at v_f_ = 4800 mm/min). A correlation between hardness and resultant forces cannot be drawn. For instance, FeMnAlC shows a totally different initial offset and progression to 316L, although it is of similar hardness. This may partly be attributed to the progression of tool wear during cutting. In the experiments, FeTiB and FeMnAlC were machined using the same milling tool, which showed no visible tool wear after the FeTiB. This conforms to the almost linear progression of the active forces. The machining of FeMnAlC caused significant tool wear, which led to the experiments being stopped prematurely. This results in a low determination coefficient for the linear fit. One explanation of the increased wear could be attributed to oxides that have formed due to the high temperatures in the cold spraying processes (indicated by annealing colors of the coating after spraying). However, this cannot be verified with the given experiments as FeMnAlC without annealing is not available for comparison. The 316L alloy was then machined using an unused tool. It shows a linear progression up to v_f_ = 4000 mm/min with a disproportionate increase thereafter. Forces rise from approx. 60 N to 190 N.

**Figure 6:**
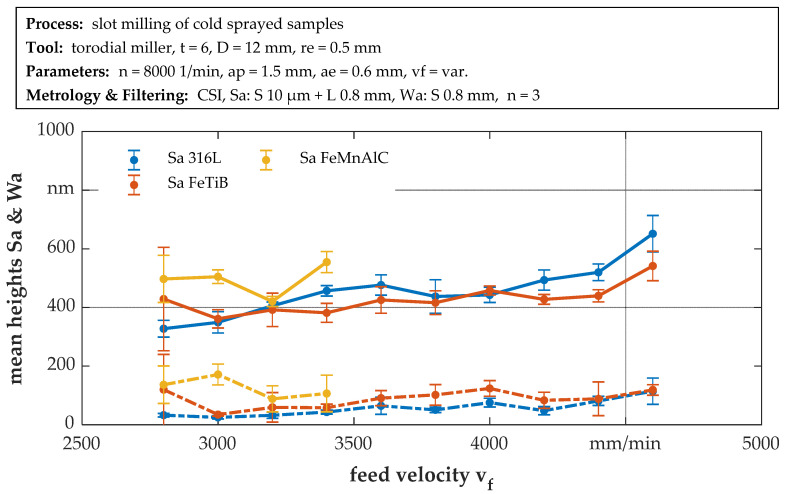
Surface mean heights Sa and Wa versus feed velocity. Error bars indicate standard deviation for repeated measurements on the same part.

The resulting surface roughness with respect to the feed velocity is assessed via the arithmetic mean height of the surface, using different filter settings. The surface roughness Sa is calculated according to ISO 25178 after applying an S-filter of 10 µm and an L-filter of 0.8 mm, while the waviness Wa (technically also “Sa”, but Wa is used here for easier differentiation) is determined using an S-filter of 0.8 mm. Average values and standard deviations are calculated across the repetitions for each feed velocity (n = 3). The results are shown in Figure 6. The graph shows that the roughness increases with increasing feed velocity for 316L and FeTiB, starting from Sa ≈ 400 nm to Sa ≈ 600 nm. Due to the small amount of applicable velocities for FeMnAlC, no definite trend can be determined in this case. The waviness values show a similar trend to the roughness values, but on a much smaller scale (Wa < 200 nm). In all cases, no fitting operation was found that yielded sufficiently high determination coefficients. Therefore, all values are connected by lines for better readability.

**Figure 7:**
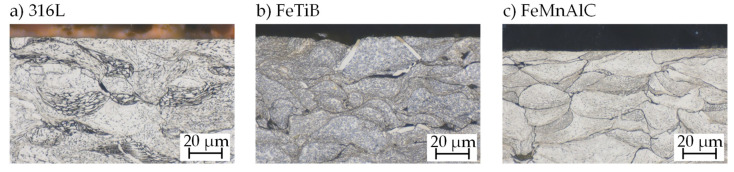
Microstructure after milling: (**a**) etched 20 s in 60% electrolytic HNO_3_ (1.5 V); (**b**,**c**) etched 15–20 s in 3% alcoholic HNO_3_.

Figure 7 shows the microstructure of all investigated materials after milling with a medium feed rate. The visible grains show the typical deformation of cold-sprayed particles, but no influence of the milling procedure is discernible: all particles are cleanly cut and show no sign of additional plastic deformation. At some locations, particles appear to have been torn from the surface; however, it cannot be clearly stated whether this is the result of the milling process, of the micrograph preparation, or of poor bonding originating in the cold spraying process.

### 3.3. Deep Rolling

**Figure 8:**
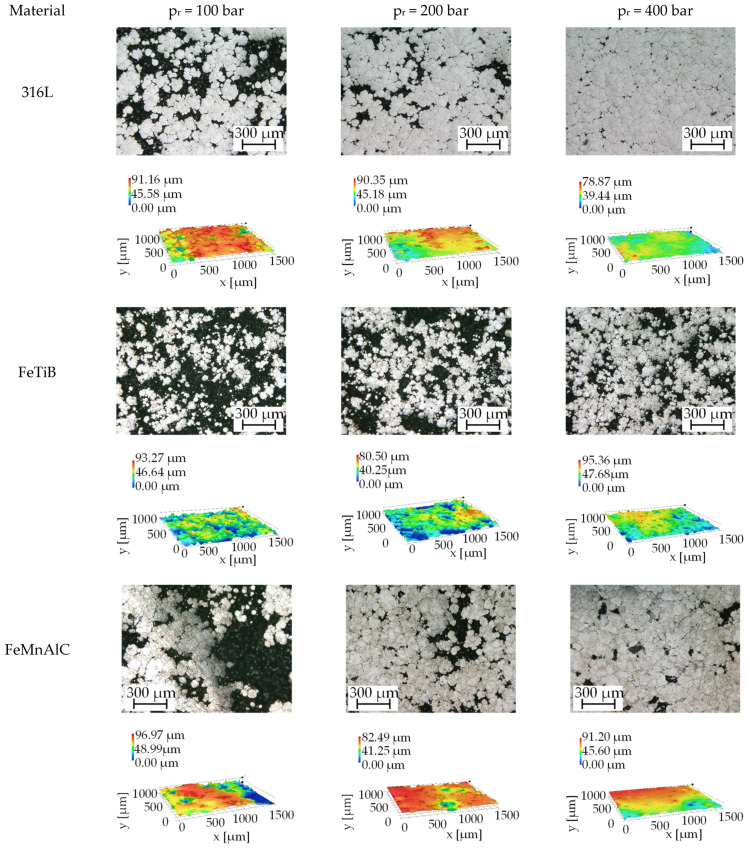
Influencing the surface topography (top view and false-color images) of cold sprayed specimens with the aid of deep rolling.

Since the samples have a high surface roughness and open porosity after cold gas spraying, the influence of deep rolling without intermediate milling is considered first. Figure 8 shows microscopic images of the sample surface (top view) and false-color images of all materials after deep rolling with varied deep rolling pressure. The surface unaffected by the rolling procedure appears dark in the microscopic images, while the part that has been deformed by the rolling is smoothed and, therefore, reflects more light and appears brighter. For all materials, the amount of deformed surface increases with rising deep rolling pressure, visible by an increasingly “closed” surface. It is assumed that the deformed particles fill the lower areas of the surface and, thereby, fill surface dents and valleys. This is more pronounced for the softer materials 316L and FeMnAlC. Due to the high initial surface roughness as well as the strong height difference of the deformed and non-deformed areas, no roughness parameters could be determined by white light interferometry for the as-sprayed states. 

**Figure 9:**
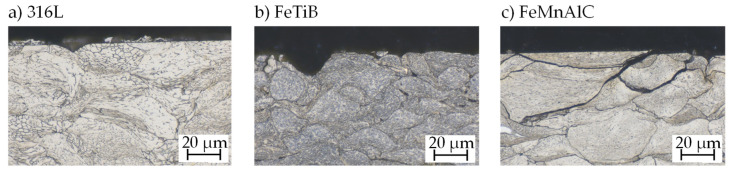
Microstructure after deep rolling with p_r_ = 400 bar (no intermediate milling): (**a**) etched 20 s in 60% electrolytic HNO_3_ (1.5 V); (**b**,**c**) etched 15–20 s in 3% alcoholic HNO_3_.

For the surface with the largest deformation (p_r_ = 400 bar), Figure 9 shows the subsurface condition with the aid of micrographs. While for 316L and FeTiB, there is no visual difference to the microstructure resulting from the cold gas spraying process, the particles deformed by the deep rolling process are clearly visible for FeMnAlC. Dark boundaries of the near-surface particles indicate cracks and that the single grains are not bonded anymore.

**Figure 10:**
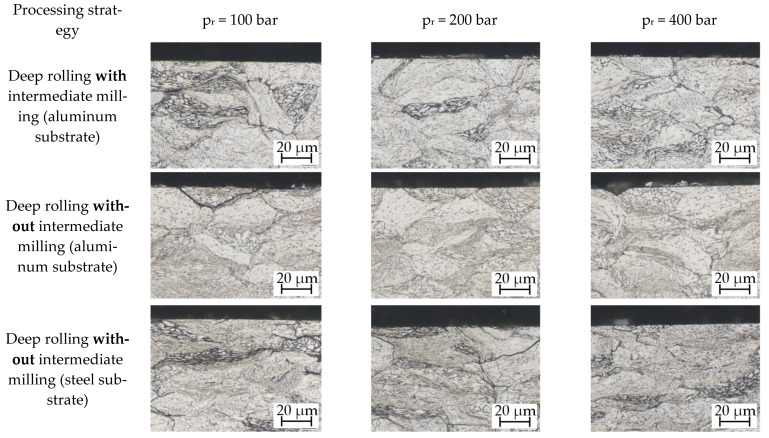
Influence of increasing deep rolling pressure with and without intermediate milling on both substrate materials for 316L (etched 20 s in 60% electrolytic HNO_3_ (1.5 V)).

In order to evaluate the influence of an increased deep rolling pressure on the subsurface properties of cold gas sprayed materials, further micrographs were generated. Figure 10 shows the microstructure obtained after deep rolling 316L samples with varied deep rolling pressure, with and without intermediate milling, on both substrate materials. There are no optical differences due to the pre-processing strategy, deep-rolling pressure, or the substrate material. The clearly visible deformation lines are distributed over the entire microstructure and result from the more significant deformation of the particles during sample generation.

To analyze the extent of the influence on the subsurface properties, hardness measurements were performed on the microsections of selected samples. Since no significant modification of the microstructure is expected or observed using the selected milling parameters, and the high surface roughness of the samples directly after the initial forming makes a comparable measurement difficult, only samples with intermediate milling are considered in the following.

**Figure 11:**
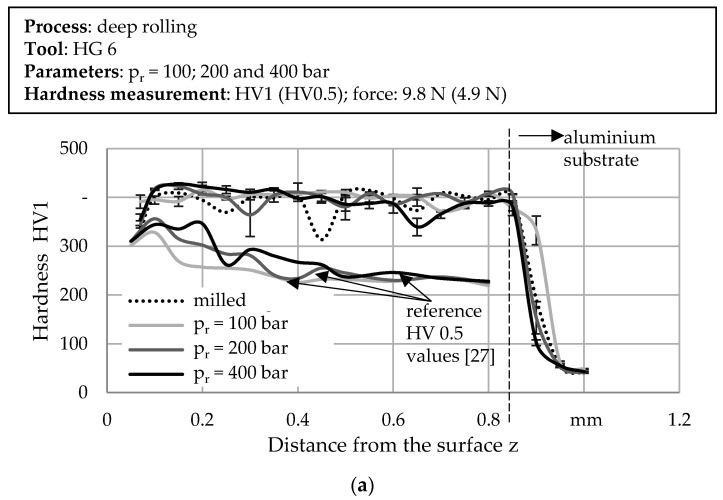

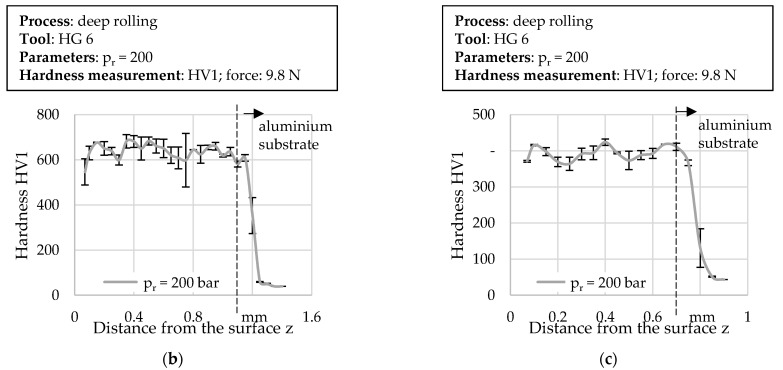
Hardness depth profiles determined (with intermediate milling) for (**a**) 316L (varied deep rolling pressure) and for a constant deep rolling pressure of 200 bar for (**b**) FeTiB and (**c**) FeMnAlC.

**Figure 12:**
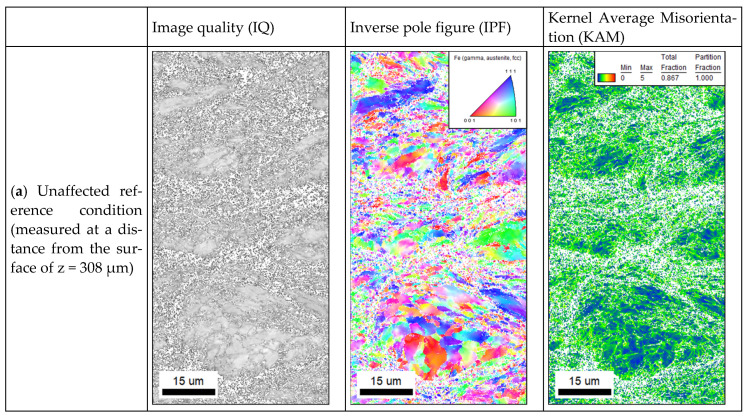

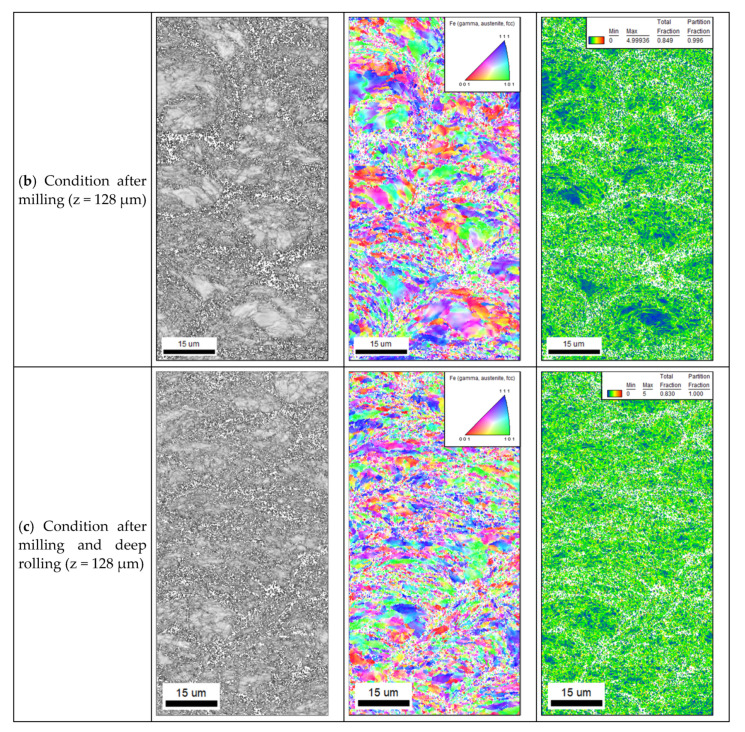
Image quality, inverse pole figure and Kernel Average Misorientation determined by EBSD measurements for (**a**) an unaffected reference condition, (**b**) a milled subsurface, and (**c**) a milled and deep rolled subsurface.

Figure 11 exemplarily shows the hardness depth profiles determined for 316L (a), FeTiB (b) and FeMnAlC (c) on aluminum substrates. Using a test load of 9.8 N and taking into account the acceptable indentation distances, it was possible to carry out two measurements in conformity with the standard below the deep rolled areas. The error bars, therefore, represent the minimum and maximum measured values determined for each depth. To investigate the influence of an increased deep rolling pressure, Figure 11a shows the hardness depth profiles for 316L with a varied deep rolling force, as well as directly after the milling process. Prior to deep rolling, the material already exhibits a hardness of about 400 HV1. This is significantly higher than the hardness of the LPBF specimens used for comparison (see: reference values). The variations visible in Figure 11 can be attributed to the microstructure. According to Liu et al., fine grains are formed at the particle–particle as well as particle–substrate interfaces [17], resulting in zones of increased hardness compared to the inner particle (cf. [7]). Applying a deep rolling pressure, regardless of its value, did not result in any visible change in the hardness level. A hardness maximum below the surface, which is characteristic for deep rolling, cannot be detected either. The hardness depth profiles of the materials FeTiB and FeMnAlC determined for a medium deep rolling pressure of 200 bar also show constant hardness levels of about 650 HV1 and 400 HV1, respectively. Since, despite visible plastic deformation, no further increase in hardness is detectable due to the deep rolling process, the latter is presumably incapable of producing further work hardening. Nevertheless, it can be assumed that additional dislocations have been formed. This is confirmed regarding the results of additionally performed EBSD measurements (see Figure 12). Image quality, inverse pole figure, and Kernel Average Misorientation (KAM) show differences between the unaffected reference condition, the condition after sole milling and the condition after milling and deep rolling. While larger areas of low misorientation (blue) are visible with and without milling, the misorientation increases after deep rolling (green). The increased number of differently colored areas occurring in the inverse pole figure after deep rolling further suggests grain refinement as a result of the process. There is no texturing visible.

## 4. Discussion

As a result of the forming process, the microstructure exhibits strongly deformed grains after cold gas spraying. In some cases, the original particles can still be recognized. Nevertheless, an evaluation of the particle compression ratio comparable to that of Liu’s work was not possible, since a large number of particles of varying size were brought to impact in order to generate the investigated coating (cf. [17]). The severe plastic deformation of the particles is accompanied by a high initial hardness. For example, the material hardness determined for 316L before deep rolling, with values of around 400 HV1, is significantly higher than the value of 240 HV0.5 reported for LPBF samples of the same material in [27].

While at a slow feed velocity of 3000 mm/min, corresponding to the material hardness, the forces are lowest for 316L, slightly higher for FeMnAlC and highest for FeTiB, no correlation between the resultant cutting force and the material hardness can be determined due to the different increases in cutting force with increasing feed velocity. The strongest increase in force is observed for FeMnAlC and the smallest for FeTiB. Considering the surface roughness, the Sa and Wa values for 316L and FeTiB increase comparably with increasing feed velocity despite significantly different hardness levels. Consequently, no correlation between the resulting variables and the material hardness could be determined so far.

Considering the influence of deep rolling, the light microscopic and false-color images of the surfaces after deep rolling already show differences due to hardness. While the surfaces of the softer materials (316L, FeMnAlC) showed increasing deformations after deep rolling with increasing deep rolling pressure, larger undeformed areas can be seen for FeTiB even at a maximum deep rolling pressure of 400 bar. On the basis of the micrographs prepared for a deep rolling pressure of 400 bar, fractured/broken out grains can be seen for FeMnAlC. Due to the high surface roughness, it was not possible to determine roughness values for the as-printed and deep-rolled without intermediate milling states by means of white-light interferometry. Consequently, a comparison with the values reported in the literature is not yet possible. For subsequent work, larger machined surface areas will, therefore, enable a tactile roughness measurement.

It can be stated that further plastic deformation of the as-sprayed surfaces occurred as a result of deep rolling. Since work hardening may thereby occur as a result of plastic deformation, comparably to the investigations of Meyer and Wielki [27], an increase in the material hardness below the surface would have been expected (see Figure 11). In contrast to this expectation, no increase in hardness could be determined as a result of the deep rolling process. This can be attributed to the high initial hardness of the material as a result of the forming. However, additional EBSD measurements showed increased misorientation and grain refinement as a result of the rolling process, confirming the hypothesis of formed dislocations, which will be addressed in subsequent studies by further analyses such as a half-width determination.

## 5. Conclusions

In this work, initial insights about the machinability of cold sprayed specimens of various materials could be gained. In both milling and deep rolling, the material properties resulting from the forming process, such as the high material hardness or particle bonding, had an influence on the process forces or the machining result. Nevertheless, no correlations between the resulting variables of both processes and the material properties could be identified so far. In subsequent work, additional analyses such as the determination of half-widths should allow further conclusions to be drawn about the material behavior.

## Figures and Tables

**Table 1:** Chemical composition in WT% of particles utilized for cold spraying (^1^ according to [28]; ^2^ according to in house measurements, cf. [29]).

	Fe	Mn	Al	C	Ti	B	Si	P	S	Cr	Ni	Mo
316L ^1^	62–68	≤2	0	≤0.03	0	0	≤1	≤0.045	≤0.03	16–18	10–14	2–3
FeTiB ^2^	82.4	0	0	0	5.6	12.0	0	0	0	0	0	0
FeMnAlC ^2^	59.1	32.7	4.6	3.6	0	0	0	0	0	0	0	0

**Table 2:** Cold gas spraying parameters.

Process gas	N_2_	
Gas pressure	50 bar	
Gas temperature	1000 °C	
Standoff distance	30 mm	
Gun travel speed	250 mm/s	
Step distance	1 mm	
Feeding rate	2 kg/h	
Nozzle path	Zigzag	
Layers	316L and FeMnAlC:	6
	FeTiB:	25 or 30
Particle size	316L:	15–38 µm
	FeTiB:	15–40 µm
	FeMnAlC:	15–40 µm

**Table 3:** Process parameter of the deep rolling process.

Component	Values		
Tool diameter d_b_ (mm)	6 (HG 6)		
Deep rolling pressure p_r_ (bar)	100	200	400
Measured rolling Force F_r_ (N)	240 ± 5	492 ± 9	996 ± 13
Step over s_o_ (mm)	0.1		
Rolling speed v_r_ (mm/min)	100		
Lubricant	8%-emulsion		
Size of deep rolled area (mm^2^)	varied ≥ 7 × 7		

## Data Availability

Data is contained within the article. Further raw data is available on request from the corresponding author. It is not publicly available since it is part of an ongoing study.

## Abbreviations

a_e_	(mm)	width of cut
a_p_	(mm)	depth of cut
d_b_	(mm)	tool diameter
F_a_	(N)	active force
F_r_, F_r, theor._	(N)	deep rolling force, theoretical deep rolling force
F_x_, F_y_	(N)	X- and Y-force components
f_peak_	(Hz)	frequency
n	(min^−1^)	spindle speed
p_r_	(bar)	deep rolling pressure
R^2^	(–)	determination coefficient
Ra, Rz	(µm)	surface roughness (profile-based)
Sa, Sz	(µm)	surface roughness (areal)
s_Fa_	(N)	standard deviation of the mean of F_a_
s_o_	(mm)	step over
v_c_	(m ∙ min^−1^)	cutting speed
v_f_	(mm/min)	feed velocity
v_r_	(mm/min)	rolling speed v_r_
Wa	(µm)	waviness
x, y, z	(µm) or (mm)	distance in different coordinate directions; z: distance from the surface
σ	(various)	standard deviation
